# Interaction of influenza A virus NS2/NEP protein with the amino-terminal part of Nup214

**DOI:** 10.3906/biy-1909-49

**Published:** 2020-04-02

**Authors:** Burçak ŞENBAŞ AKYAZI, Ayşegül PİRİNÇAL, Atsushi KAWAGUCHI, Kyosuke NAGATA, Kadir TURAN

**Affiliations:** 1 Department of Basic Pharmaceutical Sciences, Faculty of Pharmacy, Marmara University, İstanbul Turkey; 2 Department of Infection Biology, Graduate School of Comprehensive Human Sciences, University of Tsukuba, Tsukuba Japan

**Keywords:** Influenza A viruses, NS2/NEP, Nup214, nuclear pore complex, nuclear export

## Abstract

Influenza A viruses have a single-stranded RNA genome consisting of 8 segments. Each RNA segment associates with the nucleoprotein (NP) and viral RNA polymerase to and from a viral ribonucleoprotein (vRNP) particle. The viral mRNA synthesis is dependent on a capped primer derived from nascent host RNA transcripts. For these processes to take place, vRNPs must pass through the cell nuclear pore complex (NPC) to the nucleus. The influenza A virus NS2 protein, also called the nuclear export protein (NES), has an important role in the nucleocytoplasmic transport of vRNPs. This protein interacts with the host cellular nucleoporins during the nuclear export of vRNPs. In this study, the human nucleoporin 214 (Nup214) was identified as an NS2-binding protein by using a yeast two-hybrid assay. The interaction between NS2 and human Nup214 was confirmed in both yeast and mammalian cells. It has been shown that the NS2 protein interacts with the amino terminal FG domain of the Nup214 protein. The influenza viral replication was suppressed in knockdown cells for the Nup214 protein. It was concluded that the FG domains of nucleoporins have an important role in the interaction of the influenza NS2 protein with host NPC for vRNA export.

## 1. Introduction

Influenza A viruses are members of the family *Orthomyxoviridae*, which includes enveloped and segmented negative-sense single-stranded RNA (ssRNA–) viruses. The viral genome consists of 8 ssRNA molecules (Eisfeld et al., 2015; McGeoch et al., 1976). Each individual viral RNA (vRNA) is associated with the nucleoprotein (NP) as a viral ribonucleoprotein (vRNP) particle (Eisfeld et al., 2015; Honda et al., 1988; Yamanaka et al., 1990). Although the vRNA synthesis is a primer-independent process involving a complementary RNA (cRNA) intermediate, the viral mRNA synthesis is dependent on a 5′-terminal 7-methylguanosine (m7G) cap structure of host mRNAs as a primer (Pflug et al., 2017; Plotch et al., 1981). The primary dependence of influenza virus transcription differentiates these viruses from most other RNA viruses which replicate in the cytoplasm. Upon infection of the host cell, influenza vRNPs transport into the nucleus, where both transcription and replication of each vRNA is carried out (Kemler et al., 1994). The viral RNA-dependent RNA polymerase (RdRp) enzyme uses the capped primer derived from host mRNA to initiate transcription (Lukarska et al., 2017; Plotch et al., 1981). In order for these processes to take place, vRNPs must pass through the cell NPC to the nucleus and then transport progeny vRNPs to the cell cytoplasm (Boulo et al., 2007; Bui et al., 1996; Martin and Helenius, 1991). 

One of the most important functions of NPC is to coordinate the bidirectional transport of macromolecules between the cytoplasm and nucleus. It is composed of approximately 30 different proteins called nucleoporins (Hoelz et al., 2011; Krull et al., 2004; Lim et al., 2008; Lin and Hoelz, 2019). These proteins are evolutionarily conserved in distant eukaryotes from yeast to human (DeGrasse et al., 2009). The basic function of the NPC is to facilitate nucleo-cytoplasmic transport while at the same time generating a barrier between the nucleus and the cytoplasm. The NPC having a∼90–100 ˚A diameter pore size allows the free passage of macromolecules up to ∼40 kDa. The larger cargoes like viral genomes with a diameter up to ∼390 ˚A require active translocation with transport proteins (Paine et al., 1975; Pante and Kann, 2002; Rabe et al., 2003). The nucleoporins, which are structural components of NPC, are often called “Nup” followed by their molecular mass. Most nucleoporins have a domain rich in Phe and Gly (FG) repeats specifically recognized by transport proteins (Hoelz et al., 2011). The transport receptor proteins bind the FG domain of nucleoporins and slide together over the NPC channel with their cargo (Lim et al., 2008; Terry et al., 2007; Terry and Wente, 2007).

Recent studies have shown that many viruses develop different strategies for importing and exporting the nucleus by interacting with nucleoporins to complete their replication process in the host cells. It has been demonstrated that Nup214, a component of the NPC, is required for nuclear import of the adenoviral genome that performs replication in the nucleus, and the amino-terminal part of this protein acts as a direct binding site for adenoviruses to the NPC (Cassany et al., 2015). Similarly, various cellular transport proteins such as Nup153 provide crucial functions for hepatitis B virus and lentiviruses to enter the nucleus (Schmitz et al., 2010; Woodward and Chow, 2010). It is known that the influenza virus NS2 protein, which is the subject of this study, has an important role in the nucleocytoplasmic transport of the vRNPs (Dou et al., 2018). The NS2 proteins perform this function in virus-infected cells by interacting with NPC components of the host cell. The interactions of NS2 protein with human nucleoporin Rab/hRIP1 and yeast nucleoporins such as yRip1, yNup1, yNup100, and yNup116 have been shown (O’Neill et al., 1998). Recently, human nucleoporin 98 (hNup98) was identified as an NS2-binding protein by using yeast two-hybrid assay (Chen et al., 2010). The results obtained from these studies show that the NS2 protein interacts with many host proteins and that there are several unknown points in the nucleocytoplasmic transport of influenza vRNPs. 

In this study, human candidate proteins were screened for interaction with the influenza NS2 bait protein by using the yeast two-hybrid method. It was aimed to reveal the role of human nucleoporin 214 (hNup214), which has been found to be related to the NS2 in yeast cells, in influenza virus replication in mammalian cells.

## 2. Materials and methods

### 2.1. Cells and viruses

Human embryonic kidney 293 (HEK293), HeLa, and Madin-Darby Canine Kidney (MDCK) cells were used in transient transfection experiments and/or viral infections. The cells were cultured in Dulbecco’s modified Eagle’s medium (DMEM) supplemented with 10% heat-inactivated fetal calf serum (Gibco, Gaithersburg, MD, USA), 100 IU/mL penicillin, 100 µg/mL streptomycin, 2 mM glutamine, and 1.5 mg/mL sodium bicarbonate at 37°C in a humidified incubator with 5% CO2. Human influenza virus A/WSN/33 (H1N1) (WSN) and avian influenza virus A/duck/Pennsylvania/10.218/84 (H5N2) (DkPen) were propagated in MDCK cells and/or specific pathogen-free chicken embryos. The viral titer was measured using a standard plaque assay or hemagglutinin assay (Turan et al., 1996).

### 2.2. RNA extraction and first-strand cDNA preparation

In order for PCR amplification of *NUP214* cDNA and/or quantitation of the related transcripts with real-time polymerase chain reaction (RT-PCR), total RNA was prepared from the cells with the RNeasyPlusMini Kit (Qiagen, Hilden, Germany). cDNAs were prepared from 500 ng total RNA derived from HeLa and/or HEK293 cells by using Moloney murine leukemia virus reverse transcriptase (ReverTraAce, Toyobo Co., Ltd., Osaka, Japan) and oligo (dT) as a primer for 60 min at 45 °C.

### 2.3. Construction of plasmid vectors

In order to construct a plasmid vector coding the bait protein for yeast two-hybrid screening, the NS2 gene of influenza A (WSN) virus was cloned into the pGBD-C1 (James et al., 1996). NS2 ORF was amplified with PCR by using corresponding phosphorylated primers (forward: TTGAATTCGGAGGATCTGGAATGGATCCAAACACTGTGTC;reverse: TTGAATTCTTAAATAAGCTGAAACGAGA) and the mammalian expression vector pCAGGS-NS2 as a template. PCR amplification was carried out with a thermostable DNA polymerase (KOD plus, Toyobo Co., Ltd., Osaka, Japan). The PCR product was digested with *EcoR*I and purified with an agarose gel extraction kit (QiaexII, Qiagen, Hilden, Germany). pGBD-C1 plasmid was also digested with *EcoR*I, dephosphorylated with shrimp alkaline phosphatase (Thermo Fisher Scientific, Waltham, MA, USA), and ligated with PCR amplified product. The resultant plasmid was designated pGBD-NS2. For expression of NS2 proteins tagged with Flag or GST in mammalian cells, the NS2 gene was cloned into pCAGGS (Niwa et al., 1991), pCAGGS-p16-Flag (Turan and Ata, 2011), and pCAGGS-GST (Sugiyama et al., 2015) plasmids. NS2, stop codon omitted NS2 (NS2–), and Flag-NS2 ORFs were amplified with PCR by using pCAGGS-NS2 as a template with phosphorylated primers. The primer pairs ATGGATCCAAACACTGTGTC (forward) and TTAAATAAGCTGAAACGAGA (reverse), ATGGATCCAAACACTGTGTC (forward) and AATAAGCTGAAACGAGAAAGTTCT (reverse), and ATGGATTATAAAGATGATGATAAAGATCCAAACAC TGTGTC (forward) and TTAAATAAGCTGAAACGAGA (reverse) were used for PCR amplification of NS2, NS2–, and Flag-NS2 ORFs, respectively. The pCAGGS-Flag-NS2 plasmid was obtained by insertion of PCR-amplified Flag-NS2 into pCAGGS digested with *Xho*I (New England Biolabs, Hitchin, UK) and blunted with a Klenow fragment (New England Biolabs, Hitchin, UK). In order to construct pCAGGS-NS2-Flag, NS2– was cloned into the pCAGGS-p16-Flag plasmid linearized with inverse PCR by using GATTATAAAGATGATGATAAATGA (forward) and GGTGGCGGCGAATTCTTTG (reverse) primers. For pCAGGS-GST-NS2, NS2 ORF was ligated with pCAGGS-GST digested with *Sma*I (Thermo Fisher Scientific, Waltham, MA, USA). pCAGGS-NS2-GST was constructed by insertion of NS– just before the ATG code of GST of pCAGGS-GST linearized with inverse PCR by using ATGGGCTCCCCTATACTAGG (forward) and GGCGGCGCGAGCTCGAGG (reverse) primers. For the construction of Nup214 expression vectors, the carboxy-terminal part of the *NUP214* gene (*NUP214ct*), consisting of 1815 bp, was amplified from HEK293 cDNA with specific phosphorylated primers: 5´-ATCATGTCCGCTGGCAGAAGCAC-3´ (forward) and 5´-ATCTAGCTTCGCCAGCCACCAA-3´ (reverse). The PCR product was purified with the agarose gel extraction kit. In order to construct pCHA-NUP214ct encoding the HA-tagged carboxy-terminal part of Nup214 protein, the*NUP214ct* cDNA was cloned into pCHA (Nagata et al., 1998), digested with *EcoR*V (New England Biolabs, Hitchin, UK), and dephosphorylated with SAP. A plasmid encoding GAL4 AD-tagged Nup214ct was constructed by cloning the *NUP214ct* fragment into a yeast-two-hybrid expression vector, pACT2 (Clontech, #638822). The PCR-amplified *NUP214ct* cDNA was ligated with pACT2 plasmid digested with *Xho*I and blunted with Klenow fragment. The resultant plasmid was designated as pACT2-NUP214ct. The oligonucleotide primers used in different stages of the study were designed by the authors with consideration for the reference sequences of the related genes. The nucleotide sequence of each plasmid was confirmed by DNA sequencing.

### 2.4. Yeast two-hybrid screening

A fresh culture of *Saccharomyces cerevisiae *strain PJ69-4A in YPAD media was transformed with plasmid pGBD-NS2 coding GAL4-BD-NS2 bait fusion by using the lithium acetate*/*polyethylene glycol (LiAc/PEG) protocol. The transformants were selected on synthetic dropout (SD) agar plates (without Trp) and checked for the NS2 gene with PCR. One of the bait colonies was grown in YPAD media and retransformed with a cDNA library derived from HEK293 cells (Clontech, #638826), and screened following the matchmaker two-hybrid system protocol. The transformants were selected on SD agar plates (without His, Leu, Ade, and Trp) for the primary screening, and then tested with the β-galactosidase assay for the second screening. The plasmids carrying the cDNAs were isolated from potential transformants and confirmed to be positive by at least 2 independent tests with a yeast plasmid DNA miniprep kit (Bio Basic, Markham, ON, Canada) according to the manufacturer’s instructions. The plasmid samples were transformed in *E. coli* DH5α and were amplified. The cDNA inserts in the plasmids were sequenced and identified with BLAST(Basic Local Alignment Search Tool) analysis.

### 2.5. Retransforming pACT2-NUP214ct into the yeast cells and checking NS2 and Nup214 interaction 

pACT2-NUP214ct plasmid coding, a fusion of GAL4-AD-Nup214ct or pACT2 (as a control), were transformed into the *S. cerevisiae *strain PJ69-4A harboring the bait plasmid pGBD-NS2 with the LiAc/PEG protocol. Double transformants were selected on SD agar plates (without Trp and Leu). A few colonies were cultured on SD-plates (without His, Leu, Ade, and Trp), and growth profiles were defined and tested for β-galactosidase activities.

### 2.6. b-galactosidase assay

Transformed yeast cells were grown in a 5-mL SD medium (without Trp/Leu or Leu) at 30°C. The cells in 500 µL of saturated culture were recovered with centrifugation and resuspended in 300 µL of Z-buffer (0.1 M sodium phosphate, pH 7.0, 10 mM KCl, 1 mM MgSO4, and 0.27% b mercaptoethanol). The cells in suspension were disintegrated with a freeze–thaw procedure repeated 5 times in liquid nitrogen. The samples were then mixed with 60 µL o-nitrophenyl-β-d-galactopyranoside (ONPG) (4 mg/mL) and incubated for 60 min at 37°C. To stop the reaction, 300 µL Na2CO3 (0.5 M) was added. The supernatants were recovered by centrifugation at 15,000 rpm for 5 min, and the absorbance of samples at 420 nm (OD420) was defined. 

### 2.7. siRNA transfection and viral infection

The small interfering RNAs targeting exon 13 (Cat./Assay ID: 1299001/HSS111907) and exon 19 (Cat./Assay ID: 1299001: HSS111908) of *NUP214* (siNUP214) were purchased from Life Technologies (Carlsbad, CA, USA). The HeLa cells (5×105) were seeded in 6-cm petri dishes and incubated under standard culture conditions for 20–24 h. The cells were transfected with 30 pmol siNUP214 (15 pmol HSS111907 + 15 pmol HSS111908) or negative control siRNA (Invitrogen, Carlsbad, CA, USA; #12935-200) with lipofectamine RNAiMAX (Thermo Fisher Scientific, Waltham, MA, USA) according to the manufacturer’s protocol and incubated for 48 h. The cells were then subcultured into 12-well plates (2×105) and 24-well plates (1×105) and incubated for 24 h. After the incubation period, the monolayer cultures in the 24-well plate were infected with human influenza (A/WSN) or avian influenza (A/DkPen) viruses at one MOI. After virus adsorption at 37°C for 30 min, the inoculum was removed, and the cells were maintained in the maintenance medium for 8 or 12 h. The monolayers were lysed in the SDS-PAGE sample buffer at the end of the incubation period and used for viral protein analysis with Western blotting. The total RNA extraction was carried out using some of the monolayer on a 12-well plate for quantitation of the*NUP 214* transcript with RT-PCR. Remaining monolayers were infected with influenza A viruses at one MOI as mentioned above and incubated for 8 h. After incubation, total RNA was extracted for quantitation of the viral transcripts. 

### 2.8. Quantitative real-time PCR analysis

Quantitation of the*NUP214* transcript and the viral mRNAs (segment 7) in the cells transfected with siRNAs was carried out with RT-PCR. Total RNA and cDNAs were prepared as mentioned above. A real-time PCR was conducted using the FastStart Universal SYBR Green Master mixer (Roche, Mannheim, Germany). The cycle conditions included an initial denaturation step at 95 °C for 10 min, followed by 45 cycles of amplification for 5 s at 95 °C, 10 s at 55–60 °C, and 20 s at 72 °C. The quantities of the *NUP214 *transcript and the viral RNAs were normalized by the amount of actin beta (ACTB). The primer sequences used in the real-time PCR were as follows. The *NUP214* transcript:ATGTCCGCTGGCAGAAGCAC (forward)/AGAGTCAGAAGTTTGCGGAG (reverse). The *ACTB* transcript:CCACACCTTCTACAATGAGC (forward)/TCATGAGGTAGTCAGTCAGG (reverse). The viral RNA (WSN): GTGATGCCCCATTCCTTGA (forward)/TACAGAGGCCATGGTCATTT (reverse). The viral RNA (DkPen): TCATCGGTGGACTTGAATGG (forward)/TCTGACTCAACTCTTCTCGC (reverse).

### 2.9. Immunoblotting

The expression levels of Nup214, Nup214ct, and viral proteins in transfected and/or virus-infected cells were analyzed with Western blotting. The cells grown in 12- or 24-well plates were lysed in an SDS-PAGE sample buffer. The proteins in lysates were separated using SDS-PAGE and transferred to a polyvinylidene difluoride (PVDF) membrane. After blocking, the membrane was exposed to the specific primary antibodies [monoclonal mouse anti-HA (Santa Cruz Biotechnology, Dallas, TX, USA; #sc-7392), monoclonal mouse antiactin (MyBioSource, San Diego, CA, USA; #u1d3e), rabbit polyclonal anti-Nup214 (Abcam, Shanghai, China; #ab70497), polyclonal rabbit anti-NS2 (Invitrogen, #PA5-32234), and anti-M1 polyclonal rabbit antisera] overnight at 4 °C and then to a horseradish peroxidase-conjugated second antibody [antimouse IgG-HRP (Invitrogen, #31420) and/or antirabbit IgG-HRP (Invitrogen, #31423)] against species-specific immunoglobulins for 45 min at room temperature. The proteins were visualized with an ECL detection kit (GE Healthcare, Milan, Italy).

### 2.10. Immunoprecipitation 

HEK293 cells were transfected with the plasmid vectors and incubated for 48 h. After incubation, the cells were harvested and lysed in buffer A containing 50 mM Tris-HCl, pH 8.0, 150 mM NaCl, 1 mM EDTA, and 0.1% NP-40. The cell lysates were clarified by centrifugation at 10,000 rpm for 5 min. After adding 5 µL monoclonal mouse anti-HA antibody, the lysates (300 µL) were incubated at 4 °C for 2 h. Antibody–antigen complexes were mixed with protein A Sepharose beads (GE Healthcare, Uppsala, Sweden) and rotated at 4 °C for a further 8 h. Protein A Sepharose beads were recovered by centrifugation and washed 3 times with buffer A. Beads were then suspended in an SDS-sample buffer, and proteins were separated by electrophoresis through a 6% or 10% polyacrylamide gel in the presence of 0.1% SDS. The proteins were transferred to a PVDF membrane and immunoblotted.

### 2.11. Immunofluorescence assay

The localization of Nup214ct and influenza NS2 proteins in transiently transfected HeLa cells were analyzed with immunofluorescence staining. The monolayers of HeLa cells on coverslips were transfected with the plasmid vectors. After a 36–40 h transfection, the cells were washed 3 times with PBS, fixed in 3% paraformaldehyde for 15 min at room temperature, permeabilized with 0.1% NP-40, washed twice with PBS, and then treated with 1% skim milk for 30 min. The cells were incubated with the primary antibodies (mouse anti-HA and/or rabbit anti-NS2) diluted in 1% skim milk for 60 min, and washed twice with 0.1% NP-40 and once with PBS. After washing, the cells were treated with 1% skim milk for 20 min once again and then stained with Alexa-488–conjugated goat antimouse IgG and/or Alexa-568–conjugated goat antirabbit IgG (at 1:300 dilutions in 1% skim) for 60 min. The nuclei of the cells were stained with DAPI. The coverslip was washed with 0.1% NP-40 and mounted in media (0.1% p-phenylendiamine and 80% glycerol), and the cells were analyzed with a laser confocal microscope (Zeiss LSM 700; Carl Zeiss AG, Oberkochen, Germany)*.*

### 2.12. In situ proximity ligation (PLA) assay

The 50%–60% confluent HeLa cells grown on coverslips were cotransfected with the plasmids coding NS2 and Nup214ct. After a 36–40 h transfection, the cells were treated with the primary antibodies (monoclonal mouse anti-HA and polyclonal rabbit anti-NS2) as mentioned above. The assay was carried out with a Duolink PLA kit (Sigma-Aldrich, St. Louis, MO, USA; #DUO92104) by following the manufacturer’s instructions. Briefly, the monolayers were washed with wash buffer A (10 mM Tris, pH 7.4, 150 mM NaCl, 0.5% Tween-20) for 10 min and treated with a mixture of plus (mouse) and minus (rabbit) PLA probes for 90 min. The monolayers were then washed 3 times with wash buffer A for 10 min and subjected to ligation. After ligation, the closed circles were amplified by means of a rolling-circle amplification using polymerase and fluorescently labeled oligonucleotides for 3 h at 37 °C. The samples were washed 3 times with wash buffer B (200 mM Tris, pH 7.5, 100 mM NaCl) for 10 min, rinsed once with 0.01× wash buffer B, mounted in mount media, and visualized with a laser confocal microscope (Zeiss LSM 700).

## 3. Results

### 3.1. Influenza A virus NS2 protein interacts with human Nup214 in yeast cells

**Figure 1:**
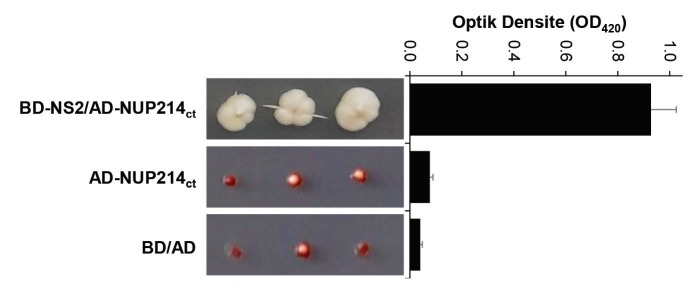
Analysis of the interaction between NS2 and Nup214ct with yeast two-hybrid assay. Left panel: growth profiles of S. cerevisiae strain PJ69-4A cells transformed with pGBD-NS2/pACT2-NUP214ct (BD-NS2/AD-Nup214ct), pACT2-NUP214ct (ADNup214 ct), or pGBD-C1/pACT2 (BD/AD) on SD-plates (without His, Leu, Ade,or Trp). Right panel: b-galactosidase activity of the yeast cells transformed with the same plasmids. AD, GAL4 activation domain. BD, GAL4 binding domain.

In order to identify the host proteins that interact with the influenza A virus NS2 protein, yeast two-hybrid screen experiments were performed by using a plasmid-coding NS2 bait and a human cDNA library cloned into pACT2. The plasmids having candidate cDNAs were isolated from the positive yeast clones, transformed in competent *Escherichia coli *DH5α, and amplified. cDNAs cloned on the plasmids were sequenced. Nucleotide sequencing revealed that one of these positive clones was harboring human *NUP214* cDNA. As a result of the sequencing, it was determined that the *NUP214* cDNA part of the *GAL4-AD-NUP214* fusion gene consisted of the sequence coding 450 amino acids residue of the carboxy-terminal part of the Nup214 protein. Therefore, a fragment consisting of 1815 nucleotides from the 3’-terminal part of the *NUP214* gene that was coding the carboxy-terminal 605 amino acid residues of Nup214 protein was amplified with RT-PCR and cloned into the pACT2 yeast two-hybrid plasmid to construct pACT2-NUP214ct. The plasmid encoding AD-Nup214ct was retransformed into the yeast cells harboring the bait plasmid, and the growth profiles of the double transformants on SD-plates (without His, Leu, Ade,or Trp) were determined (Figure 1). The results showed that the Nup214ct protein had a positive interaction with influenza A virus NS2. This interaction was also confirmed by the increase in b-galactosidase activity due to activation of the second reporter gene in the yeast cells (Figure 1).

### 3.2. Cellular nucleoporin Nup214 interacts with influenza virus NS2 protein in mammalian cells

**Figure 2:**
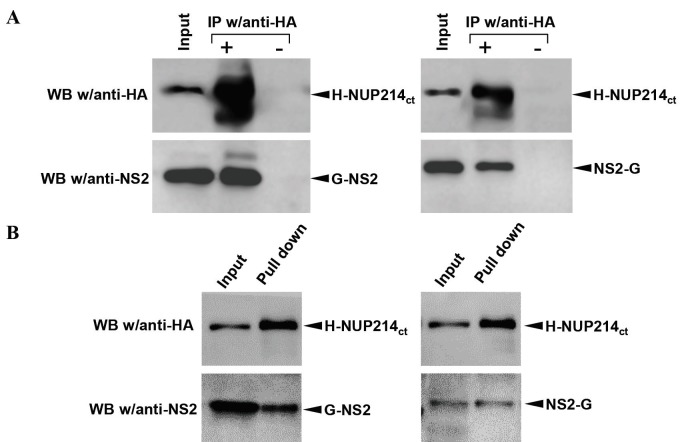
Analysis of the interaction between influenza A NS2 and human Nup214ct proteins with coprecipitation assays. The Nup214ct and GST tagged NS2 proteins expressed in HEK293 cells were precipitated with anti-HA/protein A Sepharose (a) or glutathione Sepharose (b) and then examined with SDS-PAGE/Western blotting. As the primary antibodies, mouse monoclonal anti-HA and rabbit polyclonal anti-NS2; as secondary antibody, HRP conjugated antimouse IgG or antirabbit IgG was used in Western blotting. The proteins were visualized with an ECL Western blotting detection kit (GE Healthcare).

Nucleocytoplasmic transport of viral components is a critical step for many viruses infecting human cells (Fay and Pante, 2015). It has been shown that NS2 protein and cellular nucleoporins play an important role in the nuclear export of influenza virus RNPs (Chen et al., 2010; O’Neill et al., 1998). In this study, it was shown that Nup214, one of the nucleoporin proteins, interacted with influenza NS2 protein in yeast cells by using yeast two-hybrid screening. Based on these data, the relationship between Nup214 and influenza NS2 protein was analyzed by coimmunoprecipitation, GST pull-down assay, and immunofluorescence assay in HEK293 and HeLa cells. In the first step, the possible physical interaction between Nup214 tagged with HA (H-Nup214ct) and influenza NS2 tagged with GST in 2 different orientations (G-NS2 and NS2-G) that were synthesized in transiently transfected HEK-293 cells was evaluated with coimmunoprecipitation assays. The SDS-PAGE/Western blotting analysis of precipitated protein with monoclonal mouse anti-HA antibody showed that NS2 proteins tagged with GST were coprecipitated with H-Nup214ct protein (Figure 2a). Coprecipitation tests were then carried out with glutathione Sepharose, and it was shown that the H-Nup214ct protein precipitated with the NS2 proteins (Figure 2b). These results showed that the Nup214ct and the influenza NS2 protein also interacted in transiently transfected human cells.

**Figure 3:**
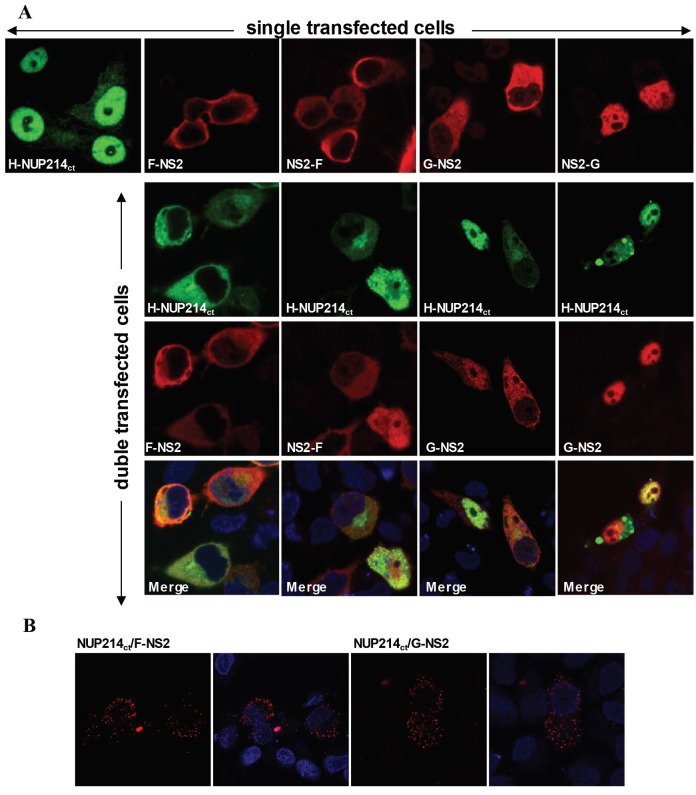
The localization patterns of influenza A virus NS2 tagged with Flag or GST and the Nup214ct proteins in the HeLa cells when expressed alone or coexpressed. At 36 h post transfection, the cells were fixed, permeabilized, and stained with mouse monoclonal anti-HA (for Nup214ct) and/or rabbit polyclonal anti-NS2 (for NS2 proteins). As the secondary antibodies, monoclonal antimouse IgG conjugated Alexa-488 (for Nup214ct), and monoclonal antirabbit IgG conjugated Alexa-568 (for NS2 proteins) were used. b. Duolink in situ proximity ligation assay. At 36 h post transfection, the cells transfected with NS2 and Nup214ct plasmids were examined with Duolink proximity l

The subcellular localization patterns of the Nup214ct protein and the NS2 proteins tagged with GST or Flag coexpressed in HeLa cells were also analyzed. Nup214ct proteins were observed in the nucleus of HeLa cells when expressed alone, suggesting that the localization was different from that of the native Nup214. Influenza NS2 proteins tagged with Flag (NS2-Flag and Flag-NS2) showed cytoplasmic localization in transiently transfected cells when synthesized alone (Figure 3). Similarly, the GST-NS2 protein was localized in the cytoplasm when expressed alone in the HeLa cells. In contrast, the NS2-GST protein was found to be located in the cell nuclei, suggesting that the orientation of the fusion proteins was important in intracellular localization (Figure 3a, upper panel). When influenza NS2 proteins carrying Flag tags and the Nup214ct protein were coexpressed in HeLa cells, the proteins showed a significantly similar localization pattern. Both proteins were dominantly localized in cytoplasm of the cells (Figure 3a). The NS2 protein carrying GST at the amino-terminal end also colocalized with Nup214ct protein, like Flag-tagged NS2 proteins. However, Nup214ct and NS2 proteins carrying GST at the carboxy-terminal end, which are cosynthesized in cells, showed a similar localization pattern in the cell nucleus. These results indicated that NS2 and Nup214 proteins closely interact and affect the localization patterns of each other under coexpression conditions in human cells. Influenza NS2 and Nup214 interactions were also examined with an in situ Duolink proximity ligation assay (PLA). After the proximity ligation reaction had taken place, fluorescent red dots were observed, supporting the results of the IP and coimmunolocalization assays (Figure 3b). 

### 3.3. NUP214 is essential for influenza replication

**Figure 4:**
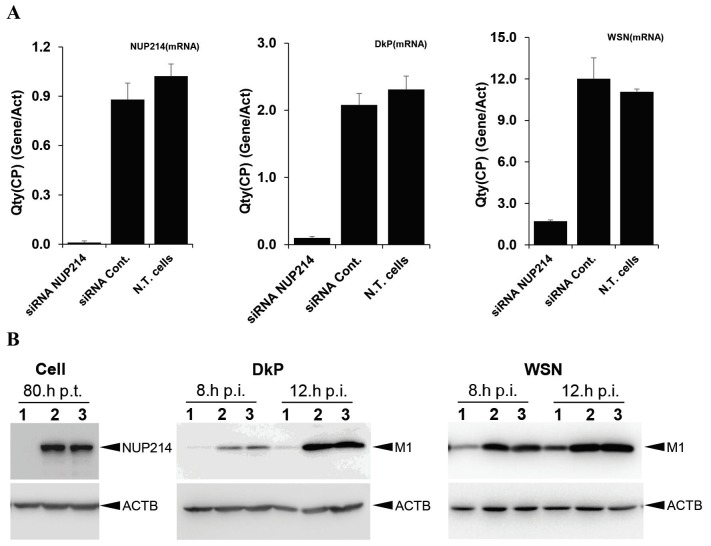
Quantitation of the viral mRNAs transcribed from segment 7 viral RNA (a) and M1 protein (b) in knockdown HeLa cells at 8 and/or 12 h p.i. The level of NUP214 mRNA and Nup214 protein in the cells just before infection were determined with quantitative real time PCR and Western blotting, respectively. The level of viral M1 mRNA was determined at 8 h p.i. For blotting of the proteins: as primary antibodies, rabbit polyclonal anti-Nup214, rabbit anti-M1 serum, and mouse monoclonal anti-ACTB were used; as secondary antibodies, HRP conjugated antirabbit IgG or antimouse IgG were used. The proteins were visualized with an ECL Western blotting detection kit (GE Healthcare).

In order to determine the effects of the Nup214 protein on influenza A virus replication, the cells were downregulated for *NUP214* with specific siRNAs (siNUP214). For this, HeLa cells were transfected with siNUP214 and negative control siRNA, as described in the method section, and then infected with human influenza A (WSN) or avian influenza A (DkPen) viruses. Immediately prior to virus infection, the levels of *NUP214* mRNA and Nup214 protein in knockdown cells were determined with RT-PCR and Western blotting, respectively. The data showed that the *NUP214* transcript level was decreased by approximately 90%. The amount of Nup214 protein decreased to an undetectable level with Western blotting under the applied conditions (Figures 4a and 4b). The total RNA was extracted from *NUP214*/knockdown cells for quantitation of viral mRNAs at 8 h postinfection. Both the WSN and DkPen mRNA levels were significantly reduced in the cells transfected with siNUP214, and the DkPen mRNA level was found to be proportionally lower than that of the WSN (Figure 4a). The levels of viral M1 protein encoded from segment 7 in infected cells at 8 and 12 h p.i. was analyzed with SDS-PAGE/Western blotting. It was shown that the levels of M1 protein dramatically decreased in parallel with the mRNA quantity (Figure 4b).

## 4. Discussion

The influenza A virus NS2 protein is a nonstructural protein encoded by RNA segment 8, the smallest segment of the viral genome. This protein carries a leucine-rich nuclear export signal and is involved in nucleo-cytoplasmic transport of vRNPs in the host cells (Neumann et al., 2000). Therefore, the NS2 is also called a nuclear export protein (NEP). Here, we screened the potential cellular proteins encoded from human cDNAs for interaction with the influenza virus NS2 protein by using a yeast two-hybrid assay, and found an interaction between the NS2 and the Nup214 proteins. The human Nup214 protein is an FG nucleoporin that is localized to the cytoplasmic side of the NPC as part of the cytoplasmic filaments (Bui et al., 2013; Paulillo et al., 2006). Structurally, this protein is composed of 3 domains: a 450-residue amino-terminal domain (NTD), 2 central coiled-coil regions that mediate the interaction with another nucleoporin protein, Nup88, and anchor Nup214 to the NPC, and a carboxy-terminal FG domain that can be detected on both sides of the NPC (Napetschnig et al., 2009). The role of the Nup214 protein in influenza A virus replication is not known. This study showed that there is a relationship between human Nup214 and NS2 protein, which is known to play a role in the nucleo-cytoplasmic transport of influenza vRNPs.

Because the DNA fragment inserted in the GAL4-AD/plasmid (pACT2-cDNA) isolated from the positive yeast colony by the yeast two-hybrid method consists of a small portion of the 3’-terminal of the *NUP214* cDNA, approximately one-third of this gene (*NUP214ct*) was recloned into the pACT2 plasmid, and a yeast two-hybrid assay was reperformed with this plasmid to confirm the interaction between influenza NS2 and the human Nup214 protein. The results showed that the coexpression of NS2 and Nup214ct induces both reporter b-galactosidase and histidine gene expression in yeast cells (Figure 1). Since there are significant structural and functional differences between yeast and mammalian cells, the relationship between viral NS2 and Nup214ct was investigated in human-originated cells (HeLa and HEK293), which are the natural host of influenza A viruses. Therefore, the interaction of the viral NS2 and Nup214ct proteins encoded from mammalian expression vectors was evaluated by coprecipitation assays, and then Western blot analyses and intracellular localization patterns using immunofluorescence techniques. The results of both the immunoprecipitation with antibodies and the GST pull-down assay revealed that the influenza A virus NS2 protein interacted with Nup214ct in human cells (Figure 2). One of the important parameters showing the relationship between the proteins is the demonstration of intracellular colocalization of proteins using immunofluorescence techniques. Therefore, intracellular localizations of GST- or Flag-tagged NS2 proteins and the Nup214ct protein consisting of the amino terminal part of the human Nup214 protein in transiently transfected HeLa cells were analyzed witha laser scanning fluorescence microscope. It was shown that the Nup214ct protein was localized within the nucleus of the cells when expressed in the absence of the NS2 protein, in contrast to the native Nup214 protein known to be localized on the cytoplasmic side of the nucleus envelope (Buiet al., 2013). The NS2 proteins tagged with the Flag and the G-NS2 protein were localized in the cell cytoplasm like the native NS2 protein (Chutiwitoonchai et al., 2014), whereas the NS2-G oriented protein was localized in the nucleus (Figure 3a, upper panel). It was observed that the Nup214ct and NS2 proteins are distinctly colocalized, and the nuclear localization pattern of the Nup214ct protein changes to the cytoplasmic location when coexpressed with NS2 in the cells. In contrast, the Nup214ct protein showed predominantly nuclear localization with the NS2-G protein in the cells when coexpressed (Figure 3a, lower panel). These results showed that the influenza A virus NS2 protein had a significant interaction with the carboxy-terminal domain of the Nup214 protein. On the other hand, the data obtained from the PLA assay for the interaction of the Flag-NS2, G-NS2, and Nup214ct proteins supported this interaction (Figure 3b).

Several studies have implied that the NS2 and M1 proteins have a role in the nuclear export of vRNPs (Martinand Helenius, 1991; Neumann et al., 2000; O’Neill et al., 1998). While the mechanism of the vRNP exports remains unclear, the current data support a model where M1 acts as an adaptor protein linking NS2 to vRNPs (Akarsu et al., 2003; Shimizu et al., 2011). It has been suggested that the NS2 protein may also interact with the CRM1 nuclear export pathway in the process of nucleo-cytoplasmic transport of influenza vRNPs (Huanget al., 2013). It is also known that the Nup214 protein is important in the CRM1-mediated export event (Fornerodet al., 1997). It has been reported that Nup214 is required for the nuclear translocation of some transcription factors through CRM1, and Nup214 binds directly to CRM1, allowing the cargo complex to be attached to the nuclear envelope (Xylourgidiset al., 2006). The mechanism of the interaction between NS2 and CRM1 in the translocation of influenza virus vRNPs and the role of the Nup214 protein in this event are not well known. It is important to answer the question of how the Nup214 protein plays a role in this phenomenon. The human Nup98 protein assessed as an FG nucleoporin like Nup214, which is the 98 kDa and is localized at the nucleoplasmic side of the NPC, has also been shown to have an interaction with the influenza NS2 protein. Chen et al. (2010) emphasized that the FG motif of hNup98 is important in the interaction with the influenza NS2 protein. This protein carries the FG motifs at the amino-terminal end (Ren et al., 2010). One of the common important properties of the Nup214 and Nup98 proteins is having FG regions that prevent the secondary structure formation of the protein. In contrast to Nup98, the Nup214 protein carries the FG motif at the carboxy-terminal part (Napetschnig et al., 2009). The data obtained with Nup214ct in this work and in previous research suggest that the affinity of the influenza NS2 protein against the FG motif may facilitate the transport of influenza A virus vRNPs by interacting directly with nucleoporins.

One of the results obtained in this work is the significant inhibition of influenza A virus transcription and translation in knockdown cells for the *NUP214* gene (Figure 4). This may be due to the effect of the Nup214 protein on host cell nucleocytoplasmic transport trafficking, as well as the inhibition of vRNP and viral mRNA translocation. The studies on the role of Nup214 in nucleo-cytoplasmic transport in mice and human cells have shown that depletion and overexpression of Nup214 either resulted in the accumulation of proteins and mRNAs in the nucleus (Boer et al., 1998; Van Deursen et al., 1996). However, the significant decreases of the influenza A virus mRNA level and viral M1 protein level in *NUP214* knockdown cells, together with the yeast two-hybrid analyses in yeast cells and its relationship with NS2 in human cells, support the conclusion that Nup214 is important for influenza viral replication.

In conclusion, here we have shown that the influenza A virus NS2 protein interacts with the amino-terminal FG domain of the Nup214 protein in yeast and human-originated cells, and influenza viral replication is suppressed in the knockdown cells for this protein. Depending on the results of this work and the previous research showing the relationship between the NS2 protein and the FG domain of the Nup98 nucleoporin, it was concluded that the FG motifs of nucleoporins may have important roles in the interaction of NS2 protein with NPC.

## Acknowledgments

The authors gratefully acknowledge helpful discussions about nucleoporins with Shoko Saito. This work was supported by a grant from the Marmara University Research Foundation (grant no.: *SAG-C-YLP-131016-0438*) and by the Scientific and Technological Research Council of Turkey (TÜBİTAK; grant no.: *SBAG-112S518*).
